# Impact of Different Group 2 Sigma Factors on Light Use Efficiency and High Salt Stress in the Cyanobacterium *Synechocystis* sp. PCC 6803

**DOI:** 10.1371/journal.pone.0063020

**Published:** 2013-04-26

**Authors:** Taina Tyystjärvi, Tuomas Huokko, Susanne Rantamäki, Esa Tyystjärvi

**Affiliations:** Molecular Plant Biology, Department of Biochemistry and Food Chemistry, University of Turku, Turku, Finland; University of South Florida College of Medicine, United States of America

## Abstract

Sigma factors of RNA polymerase recognize promoters and have a central role in controlling transcription initiation and acclimation to changing environmental conditions. The cyanobacterium *Synechocystis* sp. PCC 6803 encodes four non-essential group 2 sigma factors, SigB, SigC, SigD and SigE that closely resemble the essential SigA factor. Three out of four group 2 sigma factors were simultaneously inactivated and acclimation responses of the triple inactivation strains were studied. All triple inactivation strains grew slowly in low light, and our analysis suggests that the reason is a reduced capacity to adjust the perception of light. Simultaneous inactivation of SigB and SigD hampered growth also in high light. SigB is the most important group 2 sigma factor for salt acclimation, and elimination of all the other group 2 sigma factors slightly improved the salt tolerance of *Synechocystis*. Presence of only SigE allowed full salt acclimation including up-regulation of *hspA* and *ggpS* genes, but more slowly than SigB. Cells with only SigD acclimated to high salt but the acclimation processes differed from those of the control strain. Presence of only SigC prevented salt acclimation.

## Introduction

Transcription initiation is an important regulatory point of gene expression, and adjustments of transcription have a key role in acclimation of cyanobacteria to different stress conditions. The main regulators of transcription initiation are the sigma (σ) subunits of the RNA polymerase. *Synechocystis* sp. PCC 6803 (hereafter *Synechocystis*) encodes nine different σ subunits [1]. Replacement of one σ subunit in the RNA polymerase with another one redirects transcription, as different σ subunits recognize different promoters with different efficiency. *Synechocystis* encodes four non-essential group 2 σ factors (SigB, SigC, SigD, SigE) that closely resemble the essential primary σ factor SigA [2]–[4]. Studies of inactivation strains of group 2 σ factors have revealed that acclimation of *Synechocystis* cells to sub-optimal conditions like high temperature [2], [5]–[8], low temperature [9], and osmotic stress [4], [10], is largely dependent on group 2 σ factors. Furthermore, group 2 σ factors, especially SigD and SigB, participate in regulation of gene expression in light-dark transitions [3], [11], [12] and in acclimation to changing light quality and quantity [13]. The SigE factor is an important regulator of sugar catabolic pathways [14], [15].

Light is a key environmental factor for cyanobacteria as it is a driving force for photosynthesis. The amount and quality of light are rapidly changing in natural environments, and cyanobacteria have evolved several mechanisms balancing both perception and usage of light. Upon transfer to high light, the light harvesting phycobilisome antenna and photosystems I (PSI) and II (PSII) are down-regulated as a long term response to reduce light harvesting efficiency and photosynthetic electron transfer capacity, respectively; for a recent review see [16]. These changes adjust the balance between light reactions and carbon fixation. To protect cells against harmful effects of excess light, protective carotenoid pigments [17], high-light inducible proteins (Hlips) [18], [19] and the iron-starvation–inducible-protein, IsiA [20] accumulate in bright light. In addition, the orange carotenoid protein dependent non-photochemical quenching thermally dissipates excess energy absorbed by phycobilisomes [21], [22], and the flavodiiron protein operon *flv4-sll0218-flv2* has been suggested to be involved in photoprotection as well [23], [24]. Furthermore, state transitions balance energy distribution between the photosystems [25]. PSII is damaged in the light (photoinhibition) and without efficient PSII repair cycle one sunny day would completely inactivate PSII and thus prevent photosynthesis [26].

*Synechocystis* is a moderately halotolerant fresh water cyanobacterium tolerating up to 1.2 M NaCl for short times [27]. Physiological responses of salt acclimation in cyanobacteria are well known but salt signaling remains less clear. Cyanobacterial cells lose water and shrink rapidly upon addition of high salt to the growth medium [28]. Thereafter, diffusion of ions like Na^+^ and Cl^−^ into cells decreases water potential, and water flows back to cells [28], [29]. A high Na^+^ content of cells inhibits cellular processes, especially photosynthesis [30] and translation [31], [32]. Exchange of toxic Na^+^ to less toxic K^+^ allows reactivation of photosynthesis, and synthesis of compatible solutes begins. The main compatible solute of *Synechocystis* is glucosylglycerol [33]–[36]. Accumulation of glucosylglycerol allows efflux of extra ions, then gene expression becomes activated again, and finally changes in gene expression lead to full acclimation to high salt [32], [37].

Several hundred genes are up- or down-regulated when cells acclimate to high salt [37], [38], the majority of them encoding proteins with an unknown function. Up-regulated genes with known functions encode proteins involved in compatible solute transport and synthesis, ion transporters, and stress proteins, heat shock proteins and high-light-inducible proteins among others [37]. The expression of the *hspA* heat shock gene is particularly highly up-regulated in high salt, and an *hspA* inactivation strain is salt sensitive [39]. Synthesis of glucosylglycerol from ADP-glucose and glycerol-3-phosphate is catalyzed by the glucosylglycerol-phosphate synthetase encoded by the *ggpS* gene and the glucosylglycerol-phosphate phosphatase encoded by the *ggpP* gene; these genes are salt-inducible, and inactivation strains show extreme salt-sensitive phenotypes [35], [36]. Overexpression of an Na^+^/H^+^ antiporter in the fresh water cyanobacterium *Synechococcus* sp. PCC 7942 confers a salt-resistant phenotype [40]. Six genes encode Na^+^/H^+^ antiporters in *Synechocystis*, the *nhaS3* gene being an essential one [41]. A reduced amount of the essential NhaS3 antiporter in a non-completely-segregated inactivation strain leads to salt-sensitive phenotype [41] but inactivation strains of the other genes studied this far have not revealed particularly salt sensitive phenotypes [42].

In the present study, we focused on the effects of group 2 σ factors on moderate changes in growth light and then on acclimation to high salt. To study the effect of a particular group 2 σ factor, we used triple inactivation strains where the other group 2 σ factors were inactivated [9]. All triple inactivation strains grew slowly in low light, and our analysis suggests that the reason is reduced capacity to adjust the perception of light. On the other hand, triple inactivation strains missing simultaneously SigB and SigD were unable to enhance growth when growth light intensity was doubled. We have previously shown that ΔsigB cells acclimate only very slowly to high salt because expression of *ggpS* and *hspA* genes is low compared to that in the control strain [10]. However, obviously other group 2 σ factors also have an impact on salt acclimation, as the single inactivation strains ΔsigC and ΔsigE grow more slowly than the control strain in high salt conditions [4]. Studies with triple inactivation strains revealed that the presence of SigE as the only group 2 σ factor allows full acclimation to high salt although much more slowly than if SigB is the only functional group 2 σ factor. Cells with SigD only can grow quite well in high salt but acclimation process differs from that of the control strain, and salt acclimation is very poor if only SigC is present. The results demonstrate that group 2 σ factors are of profound importance for the ability to acclimate to environmental challenges that can be encountered by freshwater cyanobacteria.

## Materials and Methods

### Strains and growth conditions

The glucose tolerant strain of *Synechocystis* sp. PCC 6803 [43] was used as a host strain to construct the triple inactivation strains ΔsigBCD, ΔsigBCE, ΔsigBDE, and ΔsigCDE [9] and as a control strain in all experiments. Cells were grown in BG-11 medium supplemented with Hepes-NaOH, pH 7.5 at 32°C in ambient CO_2_ under constant illumination at the photosynthetic photon flux density (PPFD) of 40 µmol m^−2^s^−1^. These conditions are referred to as standard conditions. Plates for triple inactivation strains were supplemented with 50 µg µl^−1^ kanamycin, 20 µg µl^−1^ spectinomycin, 10 µg µl^−1^ streptomycin, and 5 µg µl^−1^ chloramphenicol. For the experiment, all liquid cultures were grown without antibiotics.

For growth experiments in high salt stress, BG-11 medium was supplemented with 0.7 M NaCl, optical density at 730 nm (OD_730_) of liquid cultures was set to 0.1 and cells were grown in standard conditions. Growth was followed by measuring OD_730_ every 24 h (Nikkinen et al. 2012).

For Northern blots, cells were grown in standard BG-11 medium for three days (OD_730_ ∼1), 0.7 M NaCl was added and cells were collected after 0.5, 2, 6 and 24 h of incubation in otherwise standard conditions. For a control, RNA was isolated from cells grown in standard BG-11 medium.

### Pigment ratios

*In vivo* absorption spectra were measured with a UV-3000 spectrophotometer (Shimadzu, Japan) from 400 nm to 800 nm. The heights of the carotenoid peak at 485 nm, phycobilin peak at 625 nm and chlorophyll *a* (Chl *a*) peak at 678 nm were measured from the spectra, and the ratios of phycobilin to Chl *a* and carotenoid to Chl *a* were calculated.

### 77K fluorescence spectra

Cells (35 µg Chl ml^−1^; 50 µl samples) were frozen in liquid nitrogen directly from growth conditions, or after 5 min illumination with blue light (450 nm Corion low-pass filter), PPFD 40 µmol photons m^−2^ s^−1^. 77 K fluorescence spectra were measured with an Ocean Optics S2000 spectrometer. Excitation was done using orange light obtained from a slide projector through a 580-nm narrow-band filter (Corion). The spectra were corrected by subtracting a low background signal, smoothed with a moving median using a 2-nm window, and normalized by dividing by the peak value of PSI emission at 723 nm.

### Northern blot analyses

Isolation of total RNA was performed using the hot phenol method as described previously [44]. RNA samples (5 µg) were denatured with the hot glyoxal method and RNAs were separated with 1.2% phosphate-agarose gels using a standard procedure [45]. Gene-specific probes were amplified with PCR using primers 5′-TCATCCCTTGTGATCCTTTAC-3′ and 5′-CAGCGGAAACAATTAGCCTC-3′ for *ggpS*; 5′-GTCTCTCATTCTTTACAATC-3′ and 5′-TTAGGAAGCTGAACTTTCAC-3′ for *hspA*; 5′-TCTGGGGTGGGAACTGGT-3′ and 5′-GGCGTGTTAGTGGGGTT-3′ for *nhaS3*; and 5′-AGCGTCCGTAGGTGGTTATG-3′ and 5′-CACATACTCCACCGCTTGTG-3′ for 16S rRNA. The radioactive probes were generated using the Prime-a-gene labeling kit (Promega) and [α-^32^P] dCTP (10 mCi ml^−1^; Perkin Elmer) according to the manufacturer's instructions. All membranes were stained with methylene blue prior to hybridizations to control the intactness of RNAs, and re-probed with 16S rRNA to quantify the equal loading of the samples. Autoradiograms were scanned and quantified using the FluorChem^TM^ FC imaging system (Alpha Innotech Corporation). The amount of mRNAs in each sample was corrected according to 16S rRNA data, and the mean from three independent biological experiments were calculated.

## Results and Discussion

### Characteristics of triple inactivation strains of group 2 σ factors

**Figure 1:**
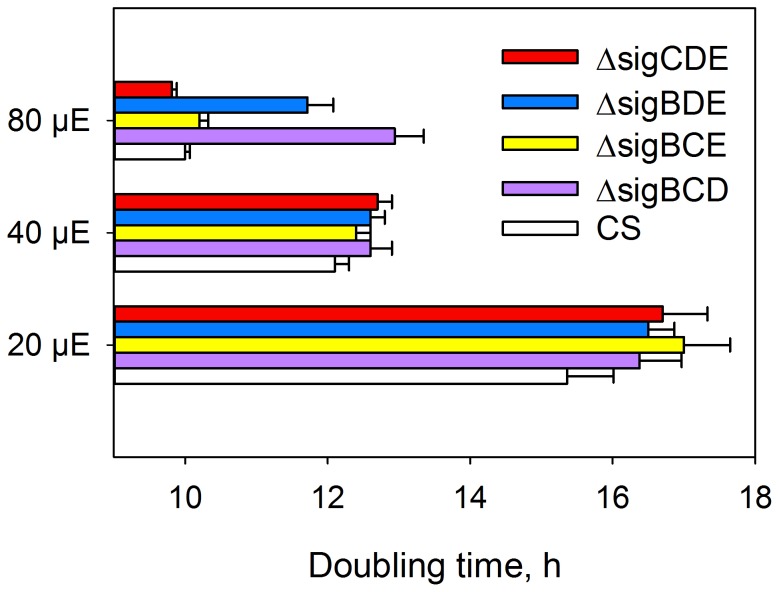
Doubling times of control (CS), ΔsigBCD, ΔsigBCE, ΔsigBDE and ΔsigCDE strains of *Synechocystis* sp.PCC 6803. Cells were grown at PPFDs of 20, 40 and 80 µmol m^−2^s^−1^ at 32°C. Each bar represents the mean of three independent biological replicates, and the error bars denote SE.

Three out of four group 2 σ factor genes were inactivated simultaneously and the resulting *Synechocystis* sp. PCC 6803 strains have only one functional group 2 σ factor, SigE in ΔsigBCD; SigD in ΔsigBCE; SigC in ΔsigBDE and SigB in ΔsigCDE [9]. All these strains grew similarly as the glucose tolerant control strain in our standard growth conditions (continuous light, photosynthetic photon flux density (PPFD) 40 µmol m^−2^s^−1^; 32°C; ambient CO_2_) having a doubling time of circa 12.5 h (Fig. 1).

**Figure 2:**
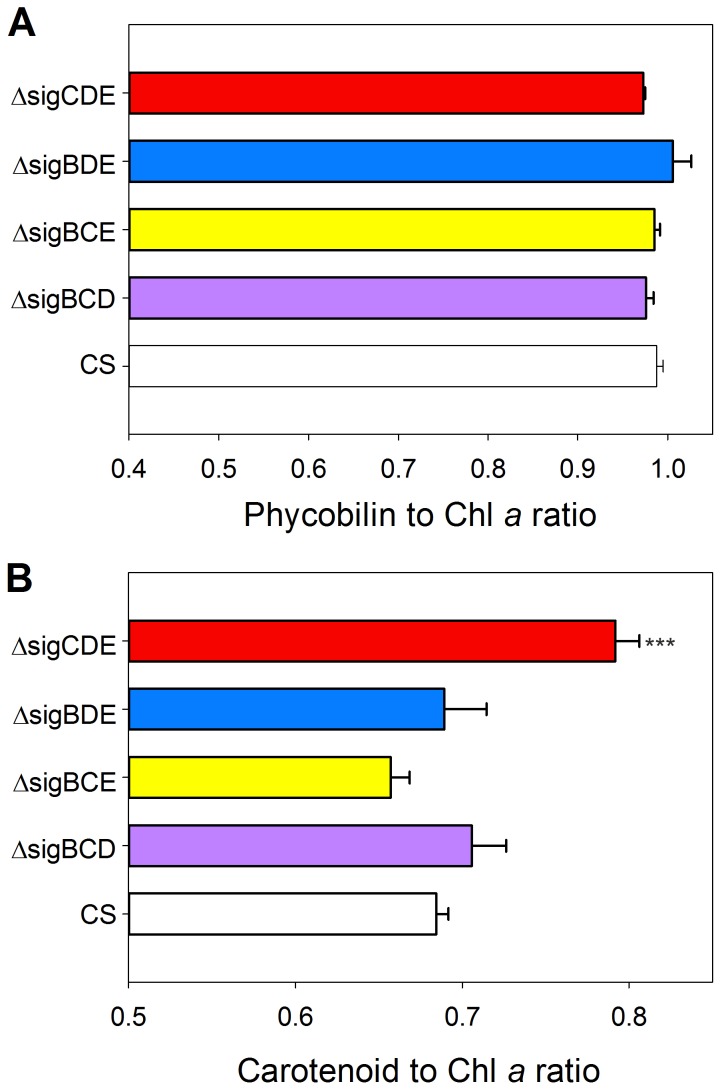
Pigment ratios of control (CS), ΔsigBCD, ΔsigBCE, ΔsigBDE and ΔsigCDE strains. The carotenoid, phycobilin and Chl *a* peaks at 485 nm, 625 nm and 678 nm, respectively, were measured from *in vivo* absorption spectra, and the ratios of phycobilin to Chl *a* (A) and carotenoids to Chl *a* (B) were calculated. Each bar represents the mean of four independent biological replicates, and the error bars denote SE. The asterisks denote a significant difference between the mutant and the control strain (P<0.001, Student's *t* test).

To evaluate the light harvesting properties of the mutant strains, the pigment composition was analysed by measuring *in vivo* absorption spectra and then calculating the phycobilin to chlorophyll (Chl) *a* ratio and the carotenoid to Chl *a* ratio (Fig. 2). The phycobilin to Chl *a* ratio of all mutant strains was similar to that measured from the control strain (Fig. 2A). The carotenoid to Chl *a* ratio, in turn, was clearly higher in the ΔsigCDE strain (1.43 fold) than in the control strain.

**Figure 3:**
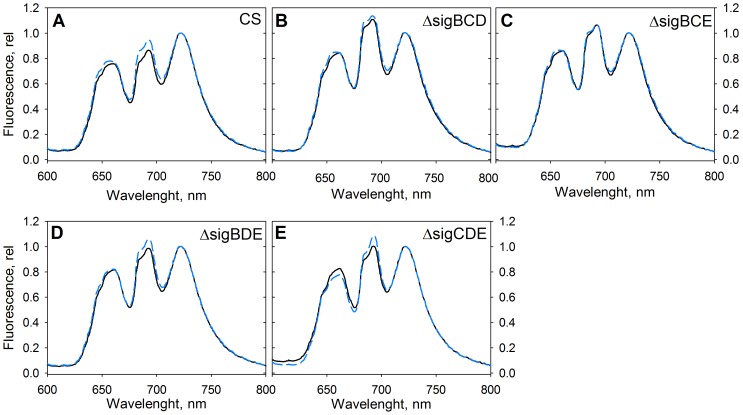
State transitions in control (A), ΔsigBCD (B), ΔsigBCE (C), ΔsigBDE (D) and ΔsigCDE (E) strains. Fluorescence was measured at 77 K with orange excitation from cells taken directly from growth conditions (grey line) or after 5 min illumination with blue light (black dashed line). The data were normalized by dividing by the height of the PSI emission peak at 723 nm. The emission peak at 640–660 nm region originate from phycobilisomes, and the PSII peaks are at 685 and 695 nm.

As the mutant strains contained normal amounts of phycobilins, we measured fluorescence emission spectra at 77 K to see if the acclimation capacity of the phycobilisome antenna differs between the strains. The control strain was in a low PSII fluorescence state (state 2) in standard growth conditions [9]. This means that the growth chamber light is more optimal to PSII than to PSI, and to balance the function of PSII and PSI, energy transfer from phycobilisomes to PSI is favoured to some extent. A 5-min treatment with blue light which is efficiently absorbed by the Chl *a* containing PSI antenna induced a high PSII fluorescence state (state 1) where light energy collected by phycobilisomes is transferred more efficiently to PSII. A clear transition from state 2 to state 1 was seen in the control strain after 5-min blue light illumination (Fig. 3A). In the control conditions, the ratio of PSII fluorescence at 685 nm and 695 nm to PSI fluorescence at 723 nm was higher in all mutant strains than in the control strain (Fig. 3), and blue light failed to induce a state 2 to state 1 transition in ΔsigBCE (Fig. 3C). According to 77 K spectra, ΔsigBCE cells were in a high PSII fluorescence state (state 1) already in standard growth conditions. The other triple inactivation strains were able to perform a state 2 to state 1 transition upon blue-light illumination, but the state transitions of the mutant strains were not as clear as in the control strain, as PSII fluorescence was high already in standard growth conditions. In fact, in the mutant strains, the ratio of PSII to PSI fluorescence always remained higher than this ratio of the control strain cells in state 1 (Fig. 3). Thus, ΔsigBCE appears to be completely locked deep in state 1, but also other group 2 σ factor inactivation strains are deeper in state 1 than can be reached by the control strain.

In dim light, at PPFD 20 µmol m^−2^s^−1^, the doubling time of the control strain was 15.4 h while the doubling times of all triple inactivation mutants were 16.5 to17 h (Fig. 1). Obviously, the triple inactivation strains were not able to acclimate to dim light as efficiently as the control strain. Interestingly, previous studies [46], [47] demonstrated that inactivation of the RpaC protein (regulator of phycobilisome association C) locks cells in state 1, and the resulting Δsll1926 strain is not able to grow in very dim light. Microarray analyses of triple inactivation strains did not detect significant differences in mRNA levels for *sll1926* (data not shown) suggesting that some other changes than a low amount of RpaC are reasons for the deep state 1 phenotype of our sigma factor mutants. However, our results support the idea that cells in state 1 do not grow well in dim light.

When the growth light was doubled (PPFD 80 µmol m^−2^s^−1^), growth of the control, ΔsigBCE and ΔsigCDE strains was enhanced, doubling times being only 10 h (Fig. 1). However, the ΔsigBCD and ΔsigBDE strains were not able to grow faster at PPFD 80 µmol m^−2^s^−1^ than in the standard growth conditions (Fig. 1). To make the most of doubled light, cells required the presence of either SigB or SigD, as these two σ factors are simultaneously missing from those strains that grew slowly in doubled light. SigB and SigD are the most similar pair of σ factors in *Synechocystis* and their functions might be partially redundant [4]. We have earlier analysed the ΔsigBD strain, and it was found in ΔsigBD that PSII and PSI centres were present in normal amounts and fully functional but the mutant strain had problems in antenna adjustments [13]. In the ΔsigBCD and ΔsigBDE strains, the phycobilin to Chl *a* ratio was similar as in the control strain (Fig. 2A) and light saturated photosynthetic and PSII activities were also similar as in the control strain [9], suggesting that problems in antenna adjustment most likely explain the slow growth of these strains in double light, just like in ΔsigBD. These results show that group 2 σ factors are not only important for acclimation to different stress conditions but also for acclimation responses that allow cells to take full advantage of environmental improvements like doubling of low growth light.

### Growth of group 2 inactivation strains in high salt

**Figure 4:**
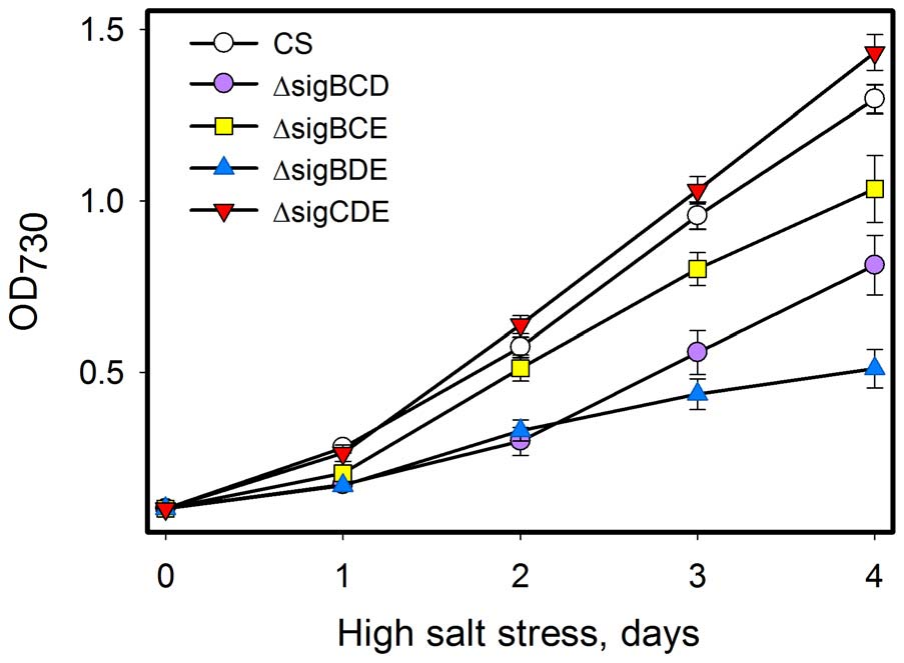
Growth of mutant strains in high salt conditions. The control (CS), ΔsigBCD, ΔsigBCE, ΔsigBDE and ΔsigCDE strains were grown in BG-11 medium supplemented with 0.7 M NaCl at 32°C under continuous light at the PPFD of 40 µmol m^−2^s^−1^.

Salt acclimation of the mutant strains was tested by growing them in BG-11 medium supplemented with 0.7 M NaCl in standard growth conditions (Fig. 4). The doubling time of the control strains at the beginning of the high salt treatment was 17 h, indicating 26% slower growth than without added salt (Fig. 4). The ΔsigCDE strain grew at least as well as the control strain, and after the first day the growth of ΔsigCDE was even slightly faster than that of the control strain. This result indicates that SigB as the only remaining group 2 σ factor is sufficient for efficient high salt acclimation of *Synechocystis*. The *sigB* gene is rapidly but only transiently up-regulated in high salt [3], [10], [37], [38]. The ΔsigB strain acclimates only slowly to high salt [4] mainly due to low expression of the *ggpS* gene involved in the synthesis of the compatible solute glucosylglycerol [10]. The salt sensitive phenotype of the ΔsigB strain can be reverted by adding compatible solutes to the growth medium [10], just like has been earlier shown for a glucosylglycerol deficient inactivation strain of the *ggpS* gene [48]. Obviously, SigB is the most important group 2 σ factor for high salt acclimation but analyses of all triple inactivation strains revealed that also other group 2 σ factors play roles in high salt acclimation.

When SigD was the only remaining group 2 σ factor (ΔsigBCE), cells grew in high salt as well as the control strain for the first two days but thereafter growth was 15% slower than in the control strain. Cells having only SigC did not properly acclimate to high-salt conditions, and the doubling time of ΔsigBDE was twice as long as in the control strain in high salt. The ΔsigBCD strain having only SigE, in turn, grew in high salt more slowly than the control strain during the first two days but thereafter growth was as fast as in the control strain, indicating that this strain was able to acclimate to high salt but the acclimation occurred more slowly than in the control strain.

### Expression of the *ggpS, hspA* and *nhaS3* genes in high salt stress

**Figure 5:**
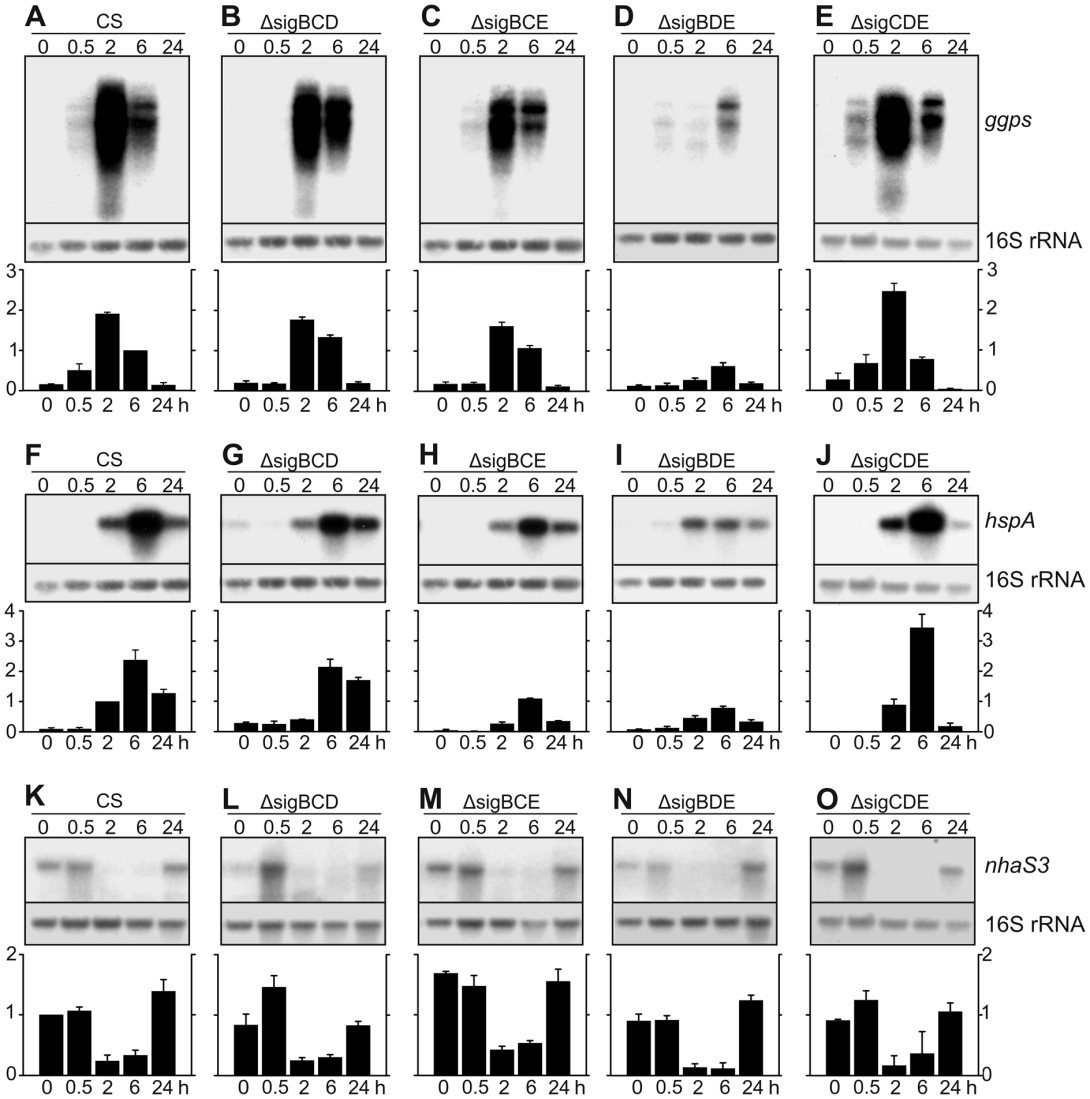
Accumulation of *ggpS, hspA* and *nhaS3* mRNAs during salt acclimation. Total RNA was isolated after 0, 0.5, 2, 6, and 24 h of treatment with 0.7 M NaCl, and the amounts of *ggpS* (A–E), *hspA* (F–J) and *nhaS3* (K–O) mRNA were determined by northern blot analysis. Samples containing 5 µg of total RNA were denaturated with glyoxal, and separated on 1.2% agarose gels in phosphate buffer. The gene specific probes were amplified by PCR and labeled with [α-^32^P] dCTP. Equal loading and even transfer of RNAs were confirmed by re-probing the membrane with a probe recognizing the16S rRNA. Representative northern blots are shown. Autoradiograms were scanned and quantified, and the amount of mRNAs in each sample was corrected according to 16S rRNA. Then data from each autoradiogram was normalized by dividing each sample by the 6-h sample of CS (*ggps*), 1-h sample of CS (*hspA*) or the 0-h sample of CS (*nhaS3*). The bars show the mean from three independent biological replicates, and the error bars denote SE.

Three aspects of salt acclimation responses were further studied by measuring the amounts of transcripts of central genes in salt acclimation. The *ggpS* gene encodes glucosylglycerol-phosphate synthase, a key enzyme in the production of the compatible solute glucosylglycerol [36]; the *hspA* gene, encoding the HspA heat shock protein that is highly upregulated in salt stress [39]; and the third gene to be studied was the essential *nhaS3* gene encoding a Na^+^/H^+^ antiporter. Representative Northern blots together with results calculated from three independent biological replicates for each gene are shown in Figure 5.

Only low levels of *ggpS* mRNAs were detected in all strains in standard conditions (Fig. 5A). Upon addition of 0.7 M NaCl, transient up-regulation of *ggpS* mRNA was observed in the control strain (Fig. 5A). The *ggpS* mRNA was clearly upregulated already 0.5 h after addition of salt, and *ggpS* mRNA content was highest in 2-h samples. In 6-h samples, the amount of *ggpS* mRNA was half of that measured after 2 h, and in samples taken after 24-h incubation in high salt, the amount of *ggpS* mRNA had returned to the same low level as measured in the standard conditions.

In ΔsigBCD and ΔsigBCE strains, up-regulation of the *ggpS* gene was not yet seen in 0.5-h samples, the highest amounts of *ggpS* mRNAs were detected in 2-h samples, *ggpS* mRNAs remained abundant in 6-h samples and only a low amount of transcripts was detected in 24-h samples (Figs. 5B and C). These results show that activation of transcription of the *ggpS* gene occurs later in ΔsigBCD and ΔsigBCE than in the control strain but the highest amounts of *ggpS* mRNA were comparable to those measured from the control strain. The ΔsigBDE strain showed a very slow induction of *ggpS* and in this strain *ggpS* transcripts remained at a very low level (Fig. 5D). Contrary to the other mutant strains, higher amounts of *ggpS* transcripts were detected in the ΔsigCDE strain than in the control strain, especially in 2-h samples (Fig. 5E). These results indicate that normal salt-induced activation of *ggpS* is dependent on the SigB factor, the presence of either SigD or SigE allows up-regulation but more slowly than in presence of SigB, and up-regulation of the *ggpS* mRNA is very poor if SigC is the only functional group 2 σ factor.

In the control strain, induction of the *hspA* gene was detected 2 h after addition of 0.7 M NaCl, the highest amount of *hspA* mRNA was detected in 6-h samples but transcripts remained abundant in the 24-h samples (Fig. 5F). In the ΔsigBCD strain, only minor up-regulation of *hspA* mRNA was seen in 2-h samples but a similar high amount of *hspA* mRNA as in the control strain was detected in the 6-h samples, and transcripts were more abundant than in the control strain in the 24-h samples (Fig. 5G). Accumulation kinetics of *hspA* mRNAs in the ΔsigBCE and ΔsigBDE resembled the kinetic pattern of the control strain but the amounts of *hspA* mRNAs remained low throughout the experiment (Figs. 5H and 5I). In the ΔsigCDE strain, in turn, a higher amount of the *hspA* mRNA was detected in 6-h samples than in the control strain (Fig. 5J). The results show that SigB is sufficient for fast and strong up-regulation of the *hspA* gene in high salt; the presence of SigE as the only group 2 σ factor allows strong but slow up-regulation; and neither SigC nor SigD can support strong expression of the *hspA* gene when present as the only group 2 σ factor. The SigB factor is required also for normal up-regulation of the *hspA* gene in high temperature stress [5], [8].

The amount of *nhaS3* mRNA in the control strain remained at the same level as in the standard growth conditions for the first 30 min in high salt. Then only low amounts of *nhaS3* transcripts were detected in the 2-h and 6-h samples, but up-regulation of *nhaS3* was detected in 24-h samples (Fig. 5K). Expression of the *nhaS3* gene in ΔsigBCD and ΔsigCDE resembled that measured in the control strain (Figs. 5K, 5L, and 5O), while slightly less *nhaS3* transcripts were detected in ΔsigBDE. In the ΔsigBCE strain, the *nhaS3* mRNAs were more abundant than in the control strain, both in the standard conditions and after 0.5-h and 24-h high salt treatments. Similar down-regulation of *nhaS3* mRNA as in the control strain occurred also in the ΔsigBCE strain (Fig. 5M). These results suggest that none of the group 2 σ factors is essential for *nhaS3* gene expression but the presence of SigD as the only group 2 σ factor enhances the expression of the *nhaS3* gene. The *nhaS3* is essential [42] but actual function of NhaS3 Na^+^/H^+^ antiporter still remains partially unclear. A reduced amount of NhaS3 leads to Na^+^ sensitivity of the cells [41]. The NhaS3 protein, however, was mainly been localized to the thylakoid membranes and thus the role of this protein in salt acclimation remains to be elucidated.

The SigB factor alone was sufficient for efficient salt acclimation, and actually ΔsigCDE grows in high salt slightly better and accumulates more *hspA* and *ggpS* transcripts than the control strain. The SigD containing strain has the second best salt acclimation capacity of the mutant strains. The SigB and SigD σ factors are the most similar σ factors in *Synechocystis* [4] but SigD does not function by replacing the regulatory function of SigB in high salt. The expression of the *ggpS* gene was slowly up-regulated in ΔsigBCE, and up-regulation of the *hsp*A gene occurred slowly and weakly. Furthermore, ΔsigBCE contained less carotenoids than the other strains (Fig. 2). Instead, the expression of the *nhaS3* gene was up-regulated in ΔsigBCE.

### Conclusions

The results shows that all group 2 triple inactivation strains show acclimation defects when growth light was only doubled or reduced to one-half, or when cells were grown in high salt stress. Our findings are summarized in Table 1. All triple inactivation strains grew slowly in low light, and our analysis suggests that the reason is a reduced capacity to adjust the perception of light. Furthermore, strains missing simultaneously SigB and SigD (ΔsigBCD and ΔsigBDE) were not able to acclimate to double light intensity and these same strains showed most severe growth defects in high salt as well (Table 1). SigB is the most important group 2 sigma factor for salt acclimation. All sigma factors compete for binding to the RNA polymerase core, and elimination of all the other group 2 sigma factors enhances the recruitment efficiency of SigB, which leads to slightly improved salt tolerance of *Synechocystis*. SigE as the only group 2 σ factor allows full salt acclimation including up-regulation of *hspA* and *ggpS* genes, but acclimation responses are slower than in the presence of SigB. Slow gene acclimation responses are accompanied with slow enhancement of growth of ΔsigBCD. Cells with only SigD acclimate to high salt quite well but cellular acclimation processes differ from those occurring in the control strain, and the presence of only SigC does not support activation of salt-induced genes leading to poor growth in high salt.

**Table 1:** Properties of group 2 σ factor triple inactivation strains in varying growth light and high salt conditions.

Strain	Group 2 σ factors	Growth, PPFD µmol m^−2^s^−1^			State transitions	Salt responses			
		20	40	80		Growth	*ggpS*	*hspA*	*nhaS3*
CS	SigB, SigC, SigD, SigE	++	+++	+++++	+++	§§§§	N n	N n	N n
ΔsigBCD	SigE	+	+++	+++	+	§§	S n	S n	N n
ΔsigBCE	SigD	+	+++	+++++	−	§§§	S n	N l	N h
ΔsigBDE	SigC	+	+++	+++	++	§	S l	N l	N n
ΔsigCDE	SigB	+	+++	+++++	++	§§§§§	F h	N h	N n

Growth of the control (CS) and triple inactivation strains at different light conditions is estimated from slow (+) to fast (+++++), and state transitions from no state transitions (−) to normal state transitions (+++). Growth at high salt conditions (§) is compared to growth of the control strain in high salt. The accumulation kinetics (Normal, N; Slow, S; Fast, F) and maximal levels of mRNAs (normal, n; low, l; high, h) are compared to those of the control strain.
